# The Mississippi Valley Dental Society

**Published:** 1870-03

**Authors:** 


### THE MISSISSIPPI VALLEY DENTAL SOCIETY.

The Mississippi Valley Dental Society held its annual meeting Wednesday, Thursday and Friday, the 3d, 4th and 5th of March.

This was one of the largest and most interesting meetings that this Society has ever held. There were about sixty members of the profession in attendance. All our neighboring States were represented.

Interesting practical subjects were before the Society, for its consideration, which elicited very spirited and thorough discussion ; and, we must say, that at no meeting of any professional society, for a long time, have we received so many new and valuable suggestions, both as respects principal and practice. In no one who attended was there manifested a want of interest, and no complaints were made that the proceedings were dull and uninstructive.

This pioneer Society, at this meeting, certainly maintained its reputation for a large amount of efficient work.

So thoroughly organized is this Society, that very little time is occupied in preliminary matters, and all the members seem to understand and feel the responsibility that rests upon them in regard to the work to be done.

And may all other associations of our profession be as efficient for good as the old Mississippi Valley Dental Society. T.

---

THOSE who will read—again and again till they understand it—the subjoined article, from the editorial columns of the *Scientific American*, will be amply repaid for their time and attention ; and they will know very much more than some of their neighbors about the philosophy of mallet force in filling teeth. Those who do not intend to read and study it, will do well to pass it by with the least possible attention. W.

